# Oligomeric epoxide–amine adducts based on 2-amino-*N*-isopropylacetamide and α-amino-ε-caprolactam: Solubility in presence of cyclodextrin and curing properties

**DOI:** 10.3762/bjoc.9.315

**Published:** 2013-12-05

**Authors:** Julian Fischer, Helmut Ritter

**Affiliations:** 1 Institute of Organic Chemistry and Macromolecular Chemistry, Heinrich-Heine-University Düsseldorf, Universitätsstraße 1, D-40225 Düsseldorf, Germany

**Keywords:** amino acids, curing properties, cyclodextrin, epoxide–amine oligomers, LCST

## Abstract

2-Amino-*N*-isopropylacetamide and α-amino-ε-caprolactam were reacted with glycerol diglycidyl ether to give novel oligomeric thermoresponsive epoxide–amine adducts. These oligomers exhibit a lower critical solution temperature (LCST) behavior in water. The solubility properties were influenced with randomly methylated β-cyclodextrin (RAMEB-CD) and the curing properties of the amine–epoxide mixtures were analyzed by oscillatory rheology and differential scanning calorimetry, whereby significant differences in setting time, viscosity, and stiffness were observed.

## Introduction

Many partially hydrophobic polymers exhibit a lower critical solution temperature (LCST) behavior in water [1–2]. Below the critical temperature (*T*_c_) these substances are completely soluble in water, whereas above the *T*_c_ they precipitate. Thereby, hydrogen bonds between polymer chains and water molecules in cold water lead to good solubility, whereas these bondings are increasingly disrupted on heating [3]. These LCST-type polymers are of interest for the development of e.g. hydrogels and drug delivery systems [2,4]. One of the most prominent LCST polymers is poly(*N*-isopropylacrylamide) (PNIPAM), which exhibits a LCST of about 32 °C [5]. The *T*_c_ can be influenced for example through copolymerization with hydrophobic or hydrophilic comonomers and further through supramolecular interactions of these comonomers with cyclodextrins (CD) [6–10]. Generally, CDs are water soluble and their ability of forming inclusion complexes with hydrophobic guest molecules is widely used in drug, food, cosmetics, textile, adhesives, plastics and more industries [11–13]. Interestingly, recently it was confirmed that α- and β-CD cannot interact with PNIPAM. Only γ-CD has a tendency to include the PNIPAM main chain [14]. Normally, epoxy resins exhibit poor solubility in water [15–17]. Accordingly, epoxide–amine polymers are not yet deeply investigated in respect to LCST behavior [18–19]. At present, most available bio-based and water soluble epoxy resins are expensive and use petroleum-based curing agents [20]. Thus, in the present paper we wish to describe our results about preparation and solubility of novel bio-based amine–epoxy oligoadducts. Also, we present some effects of CD on the solubility of these oligomers.

## Results and Discussion

**Scheme 1:**
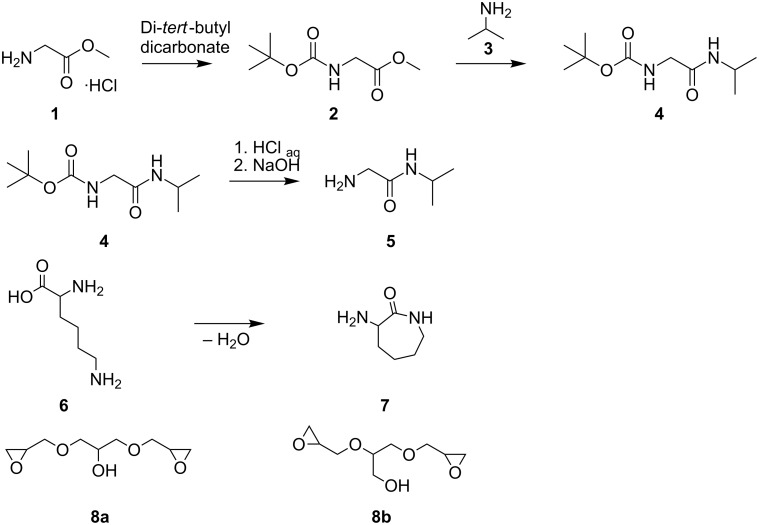
Synthesis of the monomers **5** and **7**, as well as the chemical structure of the diepoxide **8**, comprising two structural isomers **8a** and **8b**.

Generally, primary amins are bifunctional towards diepoxides and are thus monomers for the formation of corresponding oligomers [16]. Inspired by the molecular structure of *N*-isopropylacrylamide, we synthesized a primary amine 2-amino-*N*-isopropylacetamide (**5**) that is based on the amino acid glycine (Scheme 1). This was achieved in a three-step reaction through protection of the primary amino function of glycine methyl ester hydrochloride (**1**) with di-*tert*-butyl dicarbonate [21], further amidation with isopropylamine (**3**), and finally deprotection in acidic solution.

We also synthesized as a further monomer the lysine based primary amine α-amino-ε-caprolactam (**7**) through cyclization [22]. Native amino acids are not suitable for fast reaction with epoxides at low temperatures, presumably due to protonation of their amine groups that results in a reduction of their nucleophilicity [23]. However, by means of the described α-amino-ε-caprolactam (**7**), we expect to increase the reactivity of the primary amine group towards epoxides due to the formation of activating hydrogen bonds. The subsequent epoxide–amine polyaddition was carried out with glycerol diglycidyl ether (**8**), which was directly used as a mixture of the structural isomers **8a** and **8b**. To facilitate the following discussion, figures will only illustrate an idealized structure of **8**, comprising only **8a** since it contains two primary glycidyl ether groups. Ring opening oligomerization of the epoxide functions of **8** with the primary amine functions of **5** or **7**, respectively were conducted in bulk at 25 °C. The product of **5** and **8** gave oligomer **9** and that of **7** and **8** gave oligomer **10,** respectively. Thereby, to reduce branching via the hydroxy or amide groups with the epoxide functions and to obtain amine end groups, **5** and **7** were added in low excess compared to the diepoxide (molecular ratio 1.2:1 (**5**/**7**:**8**)).

**Figure 1:**
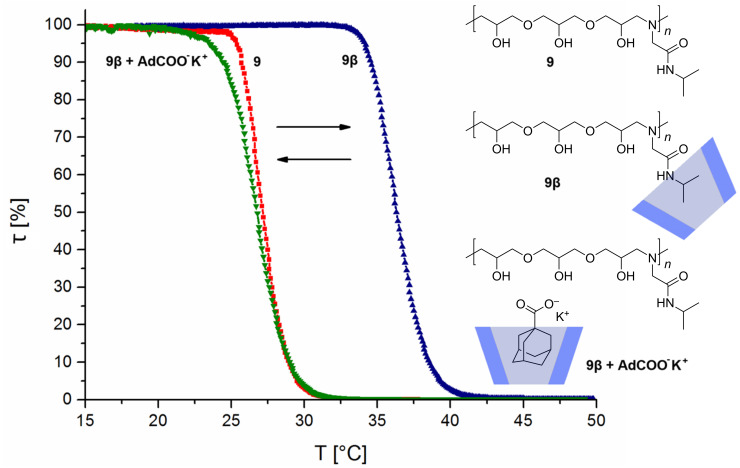
Heating curves of the LCST measurements of **9**, the complex of **9** with RAMEB-CD **9β**, and of **9** with RAMEB-CD with added AdCOO^−^K^+^.

To investigate the solution properties of **9** and **10** in water, turbidity measurements were performed (Figure 1). It was observed, that **9** was soluble in cold water and exhibited a cloud point of about 27 °C due to heating.

**Figure 2:**
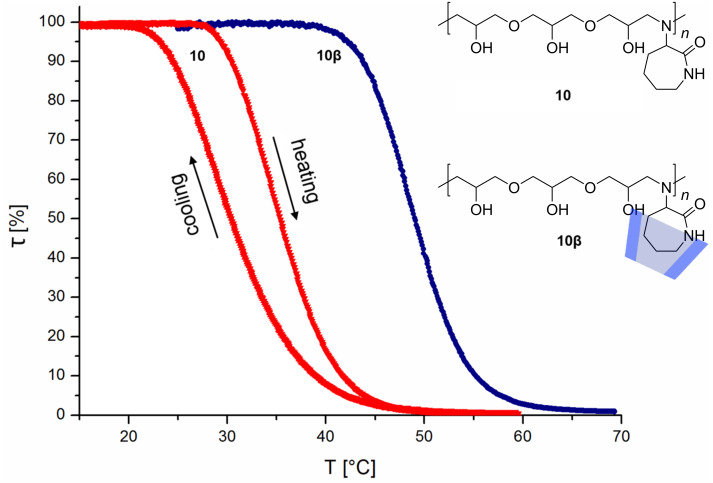
LCST measurements of **10** with illustrated heating and cooling curve, as well as the heating curve after addition of RAMEB-CD **10β**.

To evaluate the effect of CD complexation on the solubility of that oligomer **9**, 150 mol % of randomly methylated β-cyclodextrin (RAMEB-CD) were added (**9β**). After that, the cloud point shifted significantly up to 36 °C, which is remarkable since PNIPAM does not interact with β-CD [14]. Explanation for this can be steric effects, since the isopropyl group in our system **9** is placed not so close at the backbone as in PNIPAM. For further confirmation of these findings, the solution of **9** with RAMEB-CD was treated with potassium 1-adamantylcarboxylate (AdCOO^−^K^+^) in some excess, which should displace **9** from RAMEB-CD due to its high complexation properties [6,24–25]. As predicted, the cloud point shifted back to the initial temperature of about 27 °C. Oligomer **10** also exhibited LCST characteristics (Figure 2). It could be noticed that the solubility properties differ on heating and on cooling. While on heating a cloud point of about 35 °C was measured, on cooling a cloud point of about 30 °C was found. This effect is a result from insoluble to soluble transition which depends on the shape and size distribution of polymer particles.

Furthermore, **10** also showed an increase in water solubility by adding 150 mol % of RAMEB-CD **10β**. Hereby, a cloud point of about 49 °C on heating and 29 °C on cooling could be observed (Supporting Information File 1).

**Figure 3:**
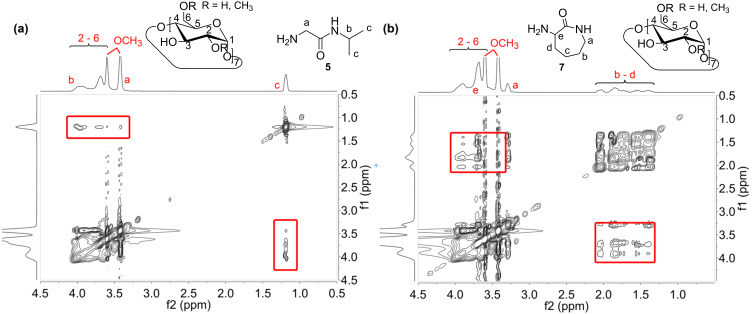
2D NMR ROESY (300 MHz, D_2_O) spectrum of the complex of **5** with RAMEB-CD (a), displaying the correlation of the isopropyl moiety with RAMEB-CD and of **7** with RAMEB-CD (b), illustrating the interaction of the lower lactam ring with RAMEB-CD.

To confirm the interaction of the isopropyl, respectively lactam moiety with RAMEB-CD we performed 2D ROESY NMR spectroscopy in D_2_O. The protons of the isopropyl group of **5** can be assigned to the proton resonances at 1.1 ppm. As illustrated in Figure 3a, these protons correlate with the protons of RAMEB-CD in the range of 3.2 ppm and 4.1 ppm. Also in case of **7**, similar findings can be observed. Hereby, the protons of the lower lactam ring between 1.1 ppm and 2.0 ppm show an interaction with the protons of RAMEB-CD (Figure 3b). 2D ROESY NMR of **9β** and **10β** were also conducted and as expected, the same correlation with RAMEB-CD as for **5** and **7** was observed (Supporting Information File 1). Unfortunately, the signals of the protons of the oligomer backbone are covered by the RAMEB-CD signals, whereby no clear correlation can be made. However, a further evidence for the self-agglomeration of RAMEB-CD with the side groups of **9** and **10** is a perceivable downfield shifting concurrent with a splitting of the concerned proton signals, since they are magnetically shielded by the CD cavity.

**Figure 4:**
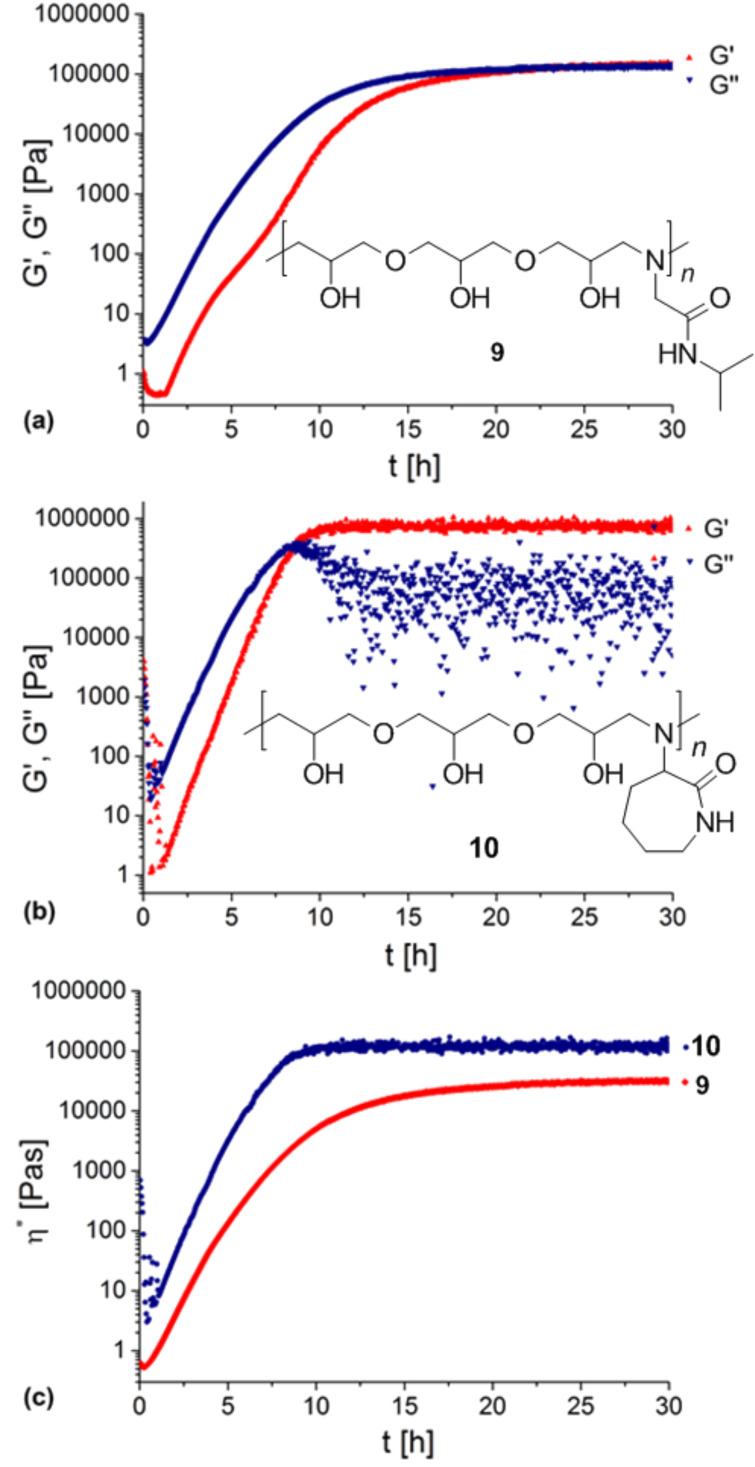
Oscillatory rheological measurements of the curing of **9** and **10**, respectively at 25 °C. Illustrated are exemplary *G’* and *G’’* plots as a function of time for **9** (a) and **10** (b), as well as a comparison of the complex viscosity of **9** and **10** (c).

**Figure 5:**
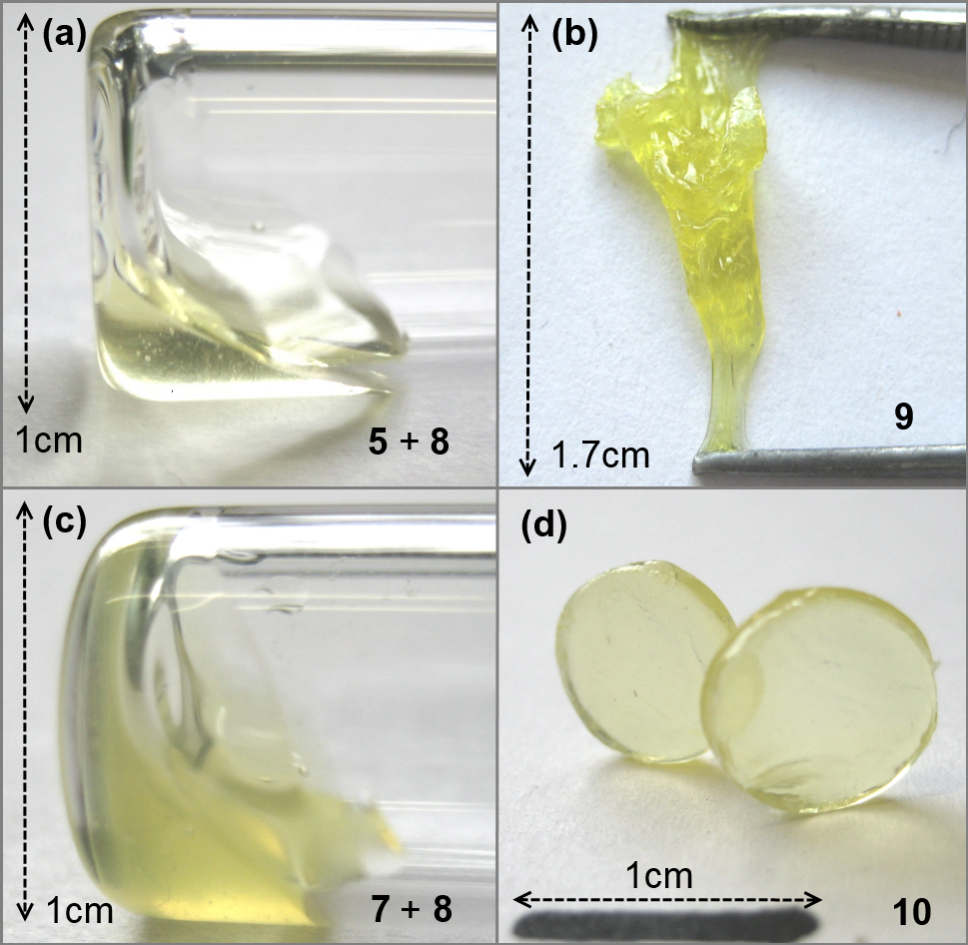
Illustration of the viscosity of a mixture of **5** and **8** (a), respectively **7** and **8** (c) before curing and the resulting materials **9** (b) and **10** (d), respectively after 24 h at 25 °C. **9** can be stretched easily by applying a force, whereas **10** can be shaped as a brittle disc.

Kinetics of the curing process at a decent temperature (25 °C) of **9** and **10** were evaluated by applying oscillatory rheological measurements. A mixture of **5** and **8** showed a low viscosity before hardening. As illustrated in Figure 4a, the mixture exhibits one-digit values for storage modulus *G’* and loss modulus *G’’* at first. With continuous reaction time the curves for *G‘* and *G‘‘* run almost parallel till they converge. It can be observed that the sol–gel transition (“gel point”), where *G‘* is equal to *G‘’*, is reached after 21 h. In theory, after this point the mixture is not capable of flowing anymore. However, after gel point *G‘* and *G‘‘* do not diverge clearly, which can be a hint for still flowable or at least flexible parts of the product. Also, horizontal lines of *G‘* and *G‘‘* shortly after reaching the gel point are a sign of a completed reaction. The resulting oligomer **9** is dimensionally stable for a certain period, but can be stretched easily by applying a force (Figure 5b).

Interestingly, a mixture of **7** and **8** yielding oligomer **10** displays 2.5-times faster curing than a mixture of **5** and **8**. In this case the gel point is reached already after about 8 to 9 h (Figure 4b). Horizontal lines for *G‘* and *G‘‘* are also observable shortly after. The resulting oligoadduct **10** can be shaped before curing and keeps this shape as a free-standing-disc after hardening (Figure 5d). This can be emphasized with the fact that *G‘* and *G‘‘* diverge considerably after the gel point transition.

A direct comparison of the curing properties of **9** and **10** is given in Figure 4c, which displays the complex viscosity (η*) calculated from *G‘* and *G‘‘.* As expected, the curves confirm the above described observations. In addition, η* at gel point transition of **10** compared to that of **9** is higher by factor of 2.8 (η* = 74 kPa*s (**10**), η* = 26 kPa*s (**9**)). The difference in rigidity of the cured products **9** and **10** is assumed to be caused by the different shape of side groups of the oligomers.

Additional proof of the different chain flexibility of the cured products **9** and **10** was conducted using DSC measurements. As expected, the *T*_g_ (glass transition temperature) of **9** (*T*_g_ = 6 °C) was significant lower than the value of the less flexible material **10** (*T*_g_ = 29 °C).

**Figure 6:**
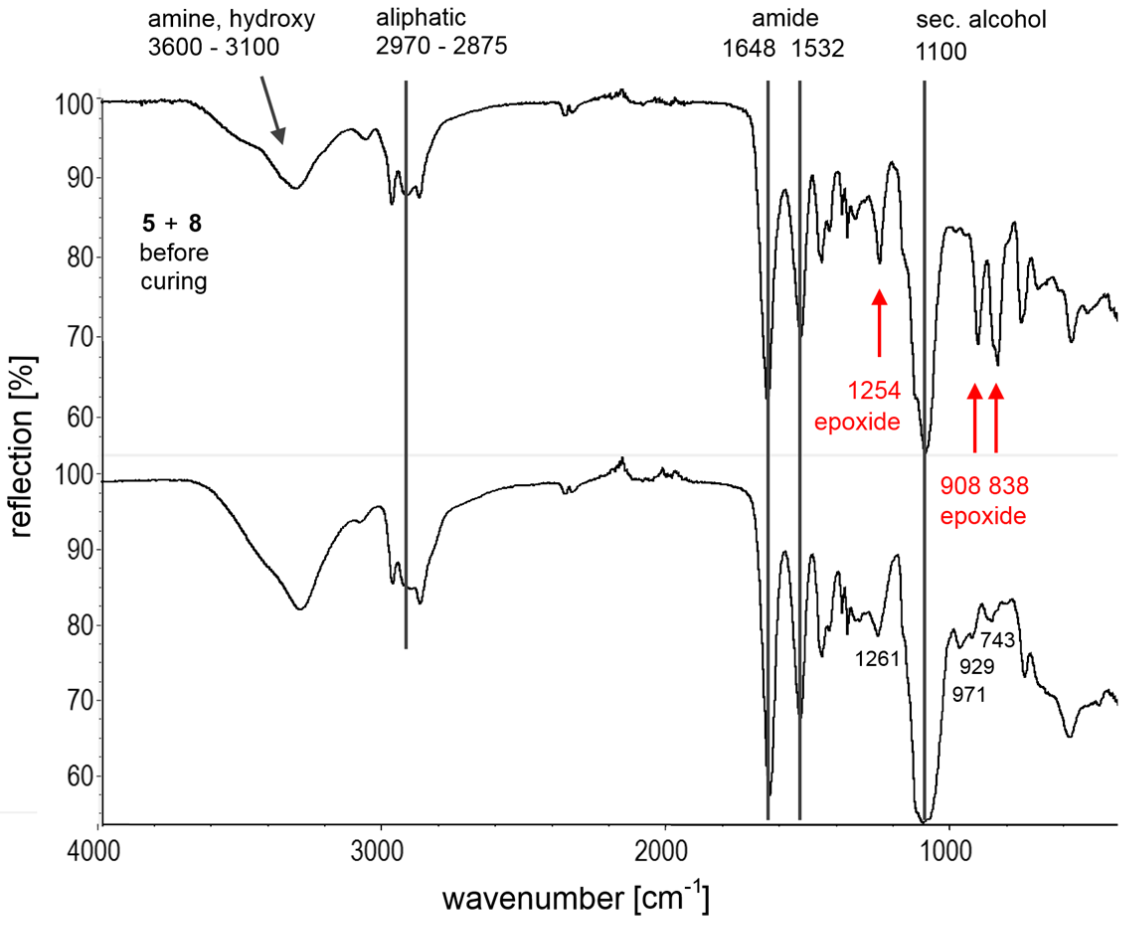
Comparison of the FTIR spectra of the monomer mixture of **5** and **8** and of the cured product **9** after 24 h.

Furthermore, FTIR spectroscopy was performed to analyze the chemical process of oligoaddition of **8** with **5** directly after mixing of the monomers and after 24 h of curing at 25 °C (Figure 6). The epoxide-function of **8** exhibits two bands at 838 cm^−1^ and 908 cm^−1^ associated with its asymmetric, respectively symmetric ring deformation vibration and one peak at 1254 cm^−1^ caused by its C–O-stretching vibration. It becomes apparent that these bands vanish after 24 h due to the complete ring opening of the former epoxide group. However, the overlapping band at 1261 cm^−1^ assigned to the amine **5** (Supporting Information File 1) complicates the analysis in this band region. Furthermore, a broadening of the bands in the region of 3100 cm^−1^ and 3600 cm^−1^ can be attributed to increasing hydrogen bonded hydroxy stretching vibrations as a result of the ring opening. Generally, these observations are a strong evidence for a high conversion of the epoxide–amine adduct.

The curing of **7** and **8** was also monitored by means of FTIR spectroscopy showing a similar disappearance of the specific bands of the epoxide function, accompanied by an increase of the hydroxy stretching vibrations (Supporting Information File 1).

Unfortunately, molecular weights of **9** and **10** could not yet be detected by use of size exclusion chromatography even under different conditions, probably due to the strong interaction of the functional groups with the column material. Also, because of its high thermosensitivity and tendency of cross linking on heating, mass spectrometry data were not useful for determination of that molecular weights. However, DLS measurements gave hydrodynamic diameters of 2.7 nm for **9** and 2.4 nm for **10** (Supporting Information File 1). This indicates the existence of oligomers rather than polymers. Comparing the hydrodynamic diameter of RAMEB-CD of ca. 1.4 nm in water (molecular weight = 1310 g/mol) with the hydrodynamic diameters of 2.7 nm for **9** and 2.4 nm for **10** these values correspond in a simple first approximation to molecular weights of around 1–3 thousands. Interestingly, the calculated values due to Carothers’ equation (1.2-fold excess of the amine, complete conversion) gave an average molecular weight of 1760 g/mol for **9** and 1826 g/mol for **10**, respectively.

## Conclusion

Two novel water soluble and thermoresponsive glycerol diglycidyl ether (**8**)–amine oligomers were obtained containing 2-amino-*N*-isopropylacetamide (**5**) and α-amino-ε-caprolactam (**7**), respectively. Complexation of RAMEB-CD with the side groups of the oligomers **9** and **10** influenced the LCST-behavior. In bulk, a mechanically flexible oligoadduct **9** and a rather brittle oligoadduct **10** were characterized.

## Experimental

All reactants were commercially available and used without further purification. All solvents used were of analytical purity or freshly distilled. RAMEB-CD was obtained from Wacker Chemie GmbH, Burghausen, Germany and were used after being dried with a vacuum oil pump over P_4_O_10_. Glycine methyl ester hydrochloride (98%) and L(+)-lysine monohydrochloride (99+%) were purchased from Acros Organics, di-*tert*-butyl dicarbonate (97+%) and isopropylamine (99+%) were obtained from Alfa Aesar, glycerol diglycidyl ether and deuterium oxide (D_2_O, 99.9% D) were purchased from Sigma Aldrich and chloroform-*d* (CDCl_3_, 99.80% D) was obtained from Euriso-Top. ^1^H NMR, ^13^C NMR and ^1^H-^1^H-ROESY spectra were recorded on a Bruker Avance DRX 300 by using deuterium oxide or chloroform-*d* as solvents. The chemical shifts (δ) are given in parts per million (ppm) using the solvent peak as an internal standard. Fourier transform infrared (FTIR) spectra were recorded on a Nicolet 6700 FTIR spectrometer equipped with an ATR unit. Turbidity measurements were conducted using a Tepper TP1 cloud-point photometer equipped with a laser with a wavelength of 670 nm. For all experiments a heating or cooling rate of 1 °C/min during continuous stirring was set and the critical temperature was determined at 50% of relative transmittance. Oscillatory rheological measurements were performed on a Haake Mars II rheometer by ThermoFisher Scientific. For this purpose a plate–plate construction (MP35, PP35Ti) was used. The temperature was set to 25 °C and was determined in the measuring plate with an accuracy of ±0.1 °C. The thermostat was regulated directly by software. For each sample the measurement period was set to 30 h. Sol–gel-transmission was determined by the arithmetic average of the first three intersections of *G’* and *G’’* taking initial fluctuations due to homogenization and sheer-thinning processes not into account. Glass transition temperatures (*T*_g_) were determined using a Mettler Toledo DSC 822e equipped with a sample robot TSO801RO. For calibration, standard tin, indium, and zinc samples were used. Heating and cooling curves were determined between −30 and 50 °C or −30 and 60 °C at a heating rate of 15 °C/min. The *T*_g_ values were calculated from the arithmetic average of the inflection points of the second, third and fourth heating curve. Electrospray ionization mass spectrometry (ESIMS) was conducted on a Bruker maXis 4G mass spectrometer and matrix-assisted laser desorption-ionization time-of-flight mass spectrometry (MALDI-TOF-MS) on a Bruker Dalomics Ultraflex 1 mass spectrometer. Melting points were obtained using a Büchi Melting Point B-545 apparatus at a heating rate of 1 °C/min. Dynamic light scattering (DLS) experiments were conducted on a Malvern Zetasizer Nano ZS ZEN 3600 at a temperature of 20 °C with a laser wavelength of 633 nm and a detection angle of 173°. The non-negative-least-squares algorithm was used for interpretation. The samples were dissolved in pure water of pH 7 and in a concentration of 1.5 mg/mL. Each measurement was performed at least five times and number-averaged diameters were used as result.

**Synthesis of 2** [21]: 8.99 g (71.6 mmol) of glycine methyl ester hydrochloride (**1**) were suspended in 60 mL methylene chloride and 6.05 g (72.0 mmol) sodium hydrogen carbonate dissolved in 80 mL water and 12 g (205.3 mmol) sodium chloride were added. Under strong stirring a solution of 15.19 g (69.5 mmol) di-*tert*-butyl dicarbonate in 40 mL of methylene chloride was poured slowly into the mixture. Subsequently, the reaction batch was refluxed for 3 h and stirred at room temperature overnight. The phases were separated, the aqueous phase was washed twice with 30 mL of methylene chloride and the organic phases were combined. After drying over magnesium sulfate and filtration thereof methylene chloride was distilled off under reduced pressure and the product dried in vacuo. 11.9 g (62.9 mmol = 87.8%) of a colorless oil was obtained. FTIR (diamond) ν (cm^−1^): 3500–3200 (br, NH), 1753 (s, ester), 1695 (vs, carbamate); ^1^H NMR (300 MHz, CDCl_3_) δ 5.22 (s, 1H, NH), 3.78 (d, *J* = 5.9 Hz, 2H, CH_2_), 3.62 (s, 3H, OCH_3_), 1.33 (s, 9H, RC(CH_3_)_3_); ^13^C NMR (75 MHz, CDCl_3_) δ 170.5 (1C, R-C(O)-OR), 155.4 (1C, RNH-C(O)-OR), 79.4 (1C, RO-C-Me_3_), 51.7 (1C, OCH_3_), 41.8 (1C, CH_2_), 27.9 (3C, RC(CH_3_)_3_); ESIMS (acetone) *m*/*z*: 190.1 [M + H]^+^, 212.1 [M + Na]^+^.

**Synthesis of 4:** 9.39 g (49.7 mmol) of **2** were mixed with 11.21 g (189.7 mmol) isopropylamine (**3**) and 5.02 g (49.7 mmol) triethylamine. The mixture was stirred at room temperature for 3 days and subsequently refluxed for 2 days. In an adjacent step the solution was concentrated and precipitated in 500 mL of *n*-hexane. After drying in vacuo 8.6 g (39.8 mmol = 80.1%) of colorless crystals (melting point 102 °C) were obtained. FTIR (diamond) ν (cm^−1^): 3292, 3222 (br, NH), 1692 (vs, carbamate), 1657, 1548 (vs, amide); ^1^H NMR (300 MHz, CDCl_3_) δ 6.41 (s, 1H, RC(O)-NH-R), 5.57 (s, 1H, R-NH-C(O)OR), 3.98 (m, 1H, CH-Me_2_), 3.66 (d, *J* = 5.7 Hz, 2H, CH_2_), 1.36 (s, 9H, RC(CH_3_)_3_), 1.06 (d, *J* = 6.6 Hz, 6H, RHC(CH_3_)_2_); ^13^C NMR (75 MHz, CDCl_3_) δ 169.1 (1C, R-C(O)-NHR), 156.7 (1C, RHN-C(O)-OR), 80.4 (1C, C-Me_3_), 44.9 (1C, CHMe_2_), 41.9 (1C, RHN-CH_2_-C(O)R), 28.8 (3C, RC(CH_3_)_3_), 23.1 (2C, RHC(CH_3_)_2_); ESIMS (acetone) *m*/*z*: 217.2 [M + H]^+^, 239.1 [M + Na]^+^.

**Synthesis of 5:** 7.56 g (35.0 mmol) of **4** were suspended in 40 mL of water. On heavy stirring 30 mL of concentrated hydrochloric acid was added dropwise. The now homogenous solution was refluxed for 3 h and stirred at room temperature overnight. Afterwards, a pH value of 12–13 of the solution was adjusted by the addition of sodium hydroxide. The solution was concentrated and subsequently lyophilized. After extracting with methylene chloride the solvent was distilled off under reduced pressure and the resulting residue was dried in vacuo to give 1.14 g (9.8 mmol = 28.1%) of a colorless oil, that turns slightly yellow on heating or after time. FTIR (diamond), ν (cm^−1^): 3600–3000 (br, NH, NH_2_), 1644, 1544 (vs, amide); ^1^H NMR (300 MHz, CDCl_3_) δ 7.08 (s, 1H, NH), 3.82 (m, 1H, CH), 3.05 (s, 2H, CH_2_), 1.49 (s, 2H, NH_2_), 0.93 (d, *J* = 6.6 Hz, 6H, RHC(CH_3_)_2_); ^13^C NMR (75 MHz, CDCl_3_) δ 171.3 (1C, C(O)), 44.5 (1C, CH_2_), 41.0 (1C, CH), 22.8 (2C, RHC(CH_3_)_2_); ESIMS (acetone) *m*/*z*: 117.1 [M + H]^+^.

**Synthesis of 7** [22]: A mixture of 20 g (109.5 mmol) L-lysine monohydrochloride (**6**), 4.38 g (109.5 mmol) sodium hydroxide, 60 g (589 mmol) aluminium oxide and 300 mL *n*-butanol is heated to reflux for 48 h in a reaction vessel equipped with a water trap. Subsequently, the mixture is filtrated and the obtained pale yellow solution is concentrated under reduced pressure. After precipitation in diethyl ether, filtration, and drying in vacuo 8.1 g (63.2 mmol = 57.7%) of colorless/pale yellow crystals (melting point = 65–69 °C) are received. FTIR (diamond), ν (cm^−1^): 3355, 3283 (br, NH), 2930, 2909, 2849 (m, CH), 1648 (s, amide); ^1^H NMR (300 MHz, D_2_O) δ 3.73 (dd, *J* = 11.0, 1.9 Hz, 1H, CH), 3.26 (m, 2H, RHN-CH_2_-R), 2.02–1.30 (m, 6H, (CH_2_)_3_); ^13^C NMR (75 MHz, D_2_O) δ 181.3 (1C, C(O)), 52.7 (1C, CH), 41.3 (1C, RHN-CH_2_-R), 32.4 (1C, RHC-CH_2_-R), 27.7 (1C, CH_2_), 27.6 (1C, CH_2_); ESIMS (H_2_O) *m*/*z*: 129.1 [M + H]^+^.

**Synthesis of 9:** The viscous monomers **5** and **8** were mixed and stirred for several minutes. The obtained pale yellow oil was kept at 25 °C without stirring for 24 h to give a sticky and highly viscous material. FTIR (diamond), ν (cm^−1^): 3600–3000 (br, NH, NH_2_, OH), 2969, 2873 (m, CH), 1641, 1534 (vs, amide), 1101 (s, COH).

**Synthesis of 10:** Monomer **7** and monomer **8** were mixed thoroughly until a homogeneous solution was achieved. Afterwards, the mixture was kept at 25 °C without stirring for 24 h to give a brittle and dimensionally stable material. FTIR (diamond), ν (cm^−1^): 3600–3000 (br, NH, NH_2_, OH), 2924, 2858 (m, CH), 1650 (s, amide), 1077 (s, COH).

## Supporting Information

The Supporting Information File 1 contains ^1^H-^1^H-ROESY spectra of oligomer **9** and **10**, respectively in presence of RAMEB-CD, additional FTIR spectra of the curing of **8** with **5** and **8** with **7**, respectively, and DLS measurements of **9** and **10**.

File 1 Additional ^1^H-^1^H-ROESY spectra, FTIR spectra and DLS data.
