# Supplementary material for: Oligomeric epoxide–amine adducts based on 2-amino-N-isopropylacetamide and α-amino-ε-caprolactam: Solubility in presence of cyclodextrin and curing properties
Source: Beilstein J Org Chem. 2013 Dec 5;9:2803–11. doi: 10.3762/bjoc.9.315 (PMC3869348; doi:10.3762/bjoc.9.315)
Supplement: File 1 — Additional 1H-1H-ROESY spectra, FTIR spectra and DLS data. [file Beilstein_J_Org_Chem-09-2803-s001.pdf]

Supporting Information

for

Oligomeric epoxide–amine adducts based on 2-amino-*N*-isopropylacetamide and α-amino-ε-caprolactam: Solubility in presence of cyclodextrin and curing properties

Julian Fischer[1] and Helmut Ritter*[1]

Address: [1]Institute of Organic Chemistry and Macromolecular Chemistry, Heinrich-Heine-University Düsseldorf, Universitätsstraße 1, D-40225 Düsseldorf, Germany

Email: Helmut Ritter - h.ritter@uni-duesseldorf.de

*Corresponding author

Additional ^1H-^1H-ROESY spectra, FTIR spectra and DLS data

Content:

- ^1H-^1H-ROESY of oligomer **9** in presence of RAMEB-CD
- ^1H-^1H-ROESY of oligomer **10** in presence of RAMEB-CD
- Extended FTIR analysis of the curing of **5** and **8**
- FTIR analysis of the curing of **7** and **8**
- Determination of the hydrodynamic diameter of **9**
- Determination of the hydrodynamic diameter of **10**
- Hysteretic effect of **10** in aqueous RAMEB-CD solution

^{1}H-^{1}H-ROESY of oligomer 9 in presence of RAMEB-CD:

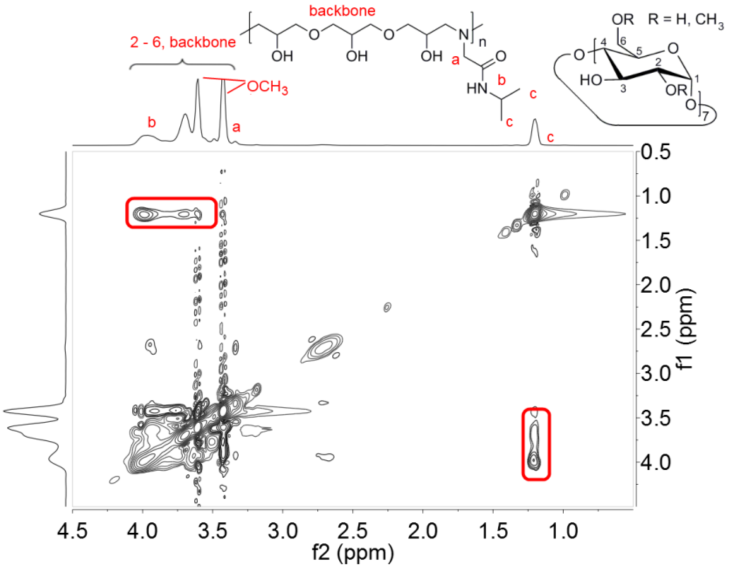

Figure S1: 2D NMR ROESY (300 MHz, D_2O) spectrum of the complex of **9** with RAMEB-CD, displaying the correlation of the protons at 1.1 ppm and the protons in the region of 3.3–4.1 ppm.

[1]H-[1]H-ROESY of oligomer 10 in presence of RAMEB-CD:

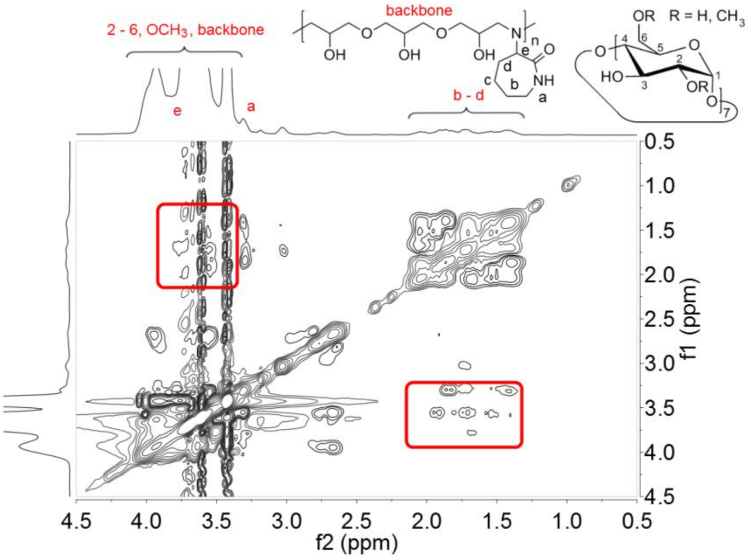

Figure S2: 2D NMR ROESY (300 MHz, D_2O) spectrum of the complex of **10** with RAMEB-CD, displaying the correlation of the protons in the region of 1.1–2.0 ppm and the protons in the region of 3.3–4.1 ppm.

Extended FTIR analysis of the curing of 5 and 8:

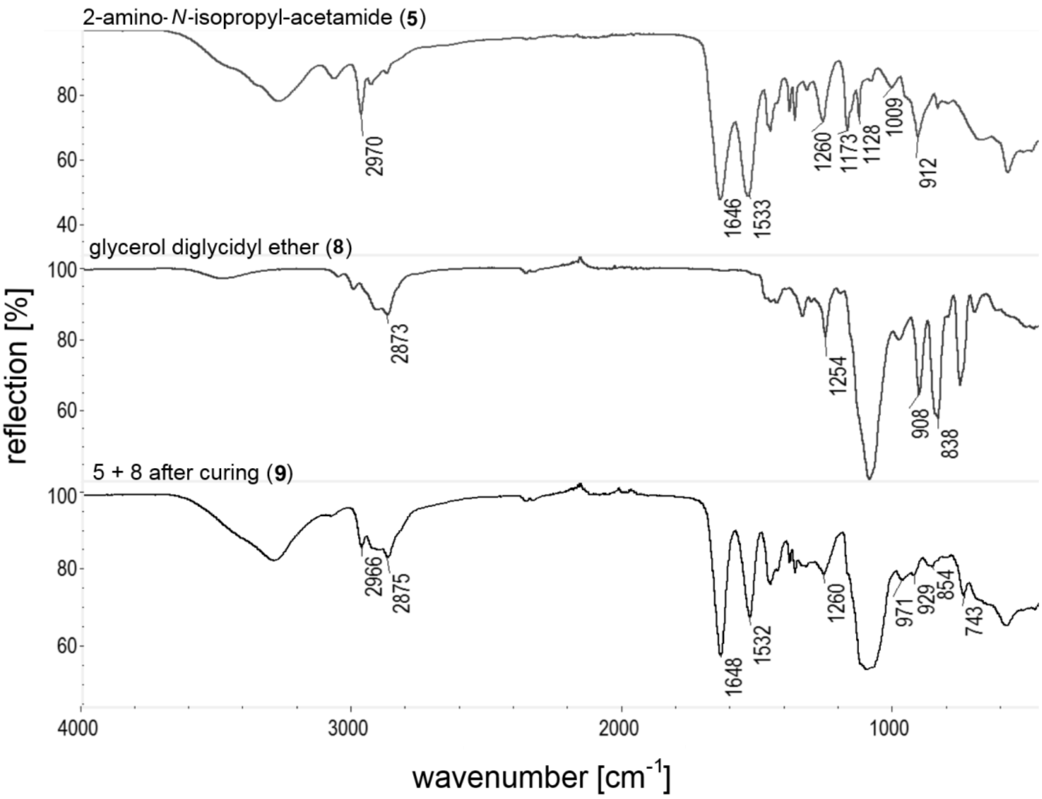

Figure S3: Comparison of the FTIR spectra of the monomers **5** and **8**, as well as the resulting adduct **9** after curing.

FTIR analysis of the curing of 7 and 8:

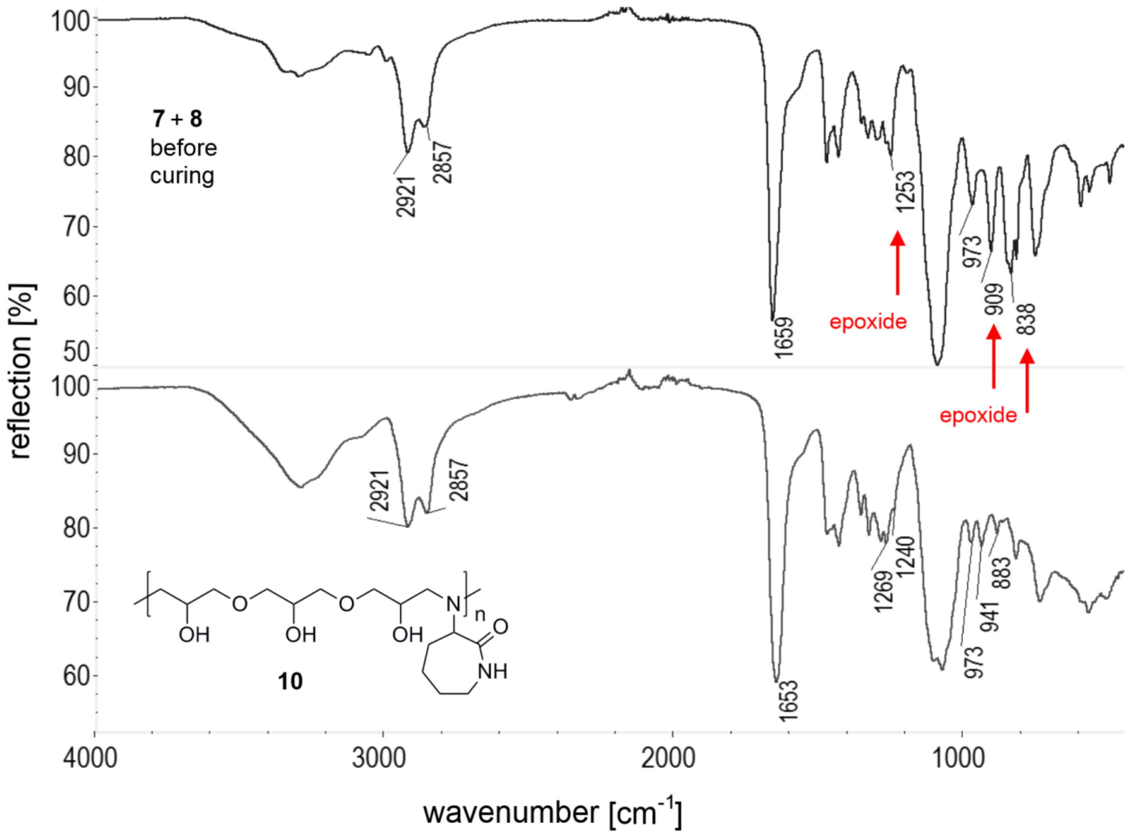

Figure S4: Comparison of the FTIR spectra of the monomer mixture of **7** and **8** and the oligomer **10** after curing.

Determination of the hydrodynamic diameter of 9:

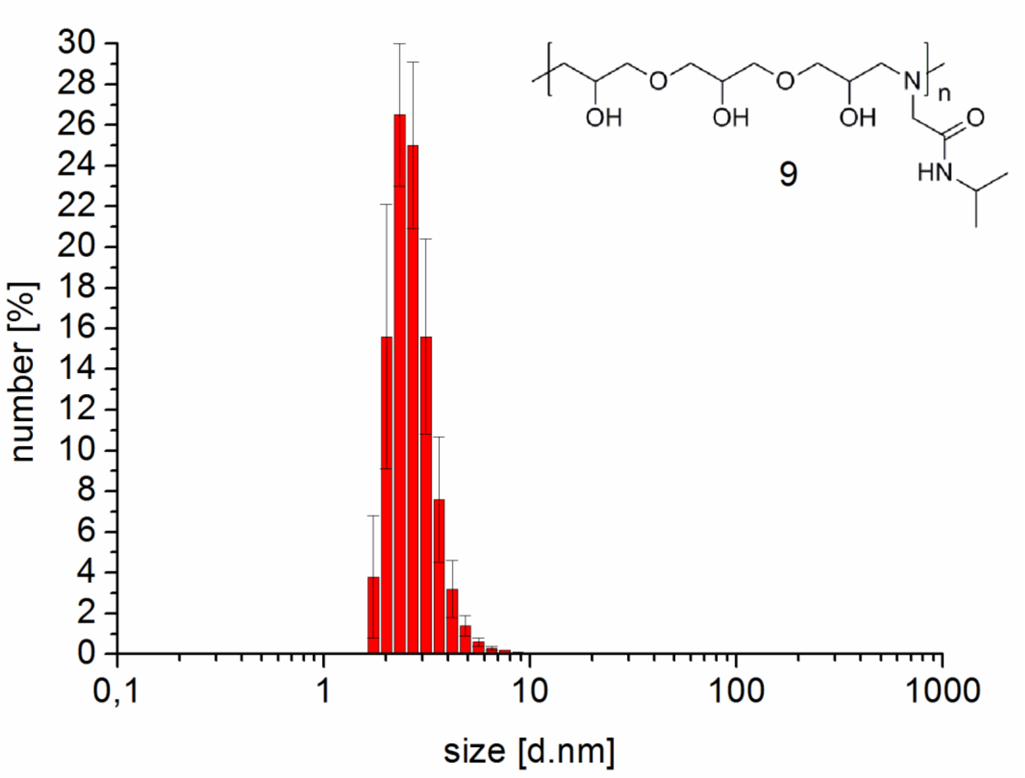

Figure S5: Mean hydrodynamic diameters of **9** in water (1.5 mg/mL) at 20 °C and standard deviation error bars.

Determination of the hydrodynamic diameter of 10:

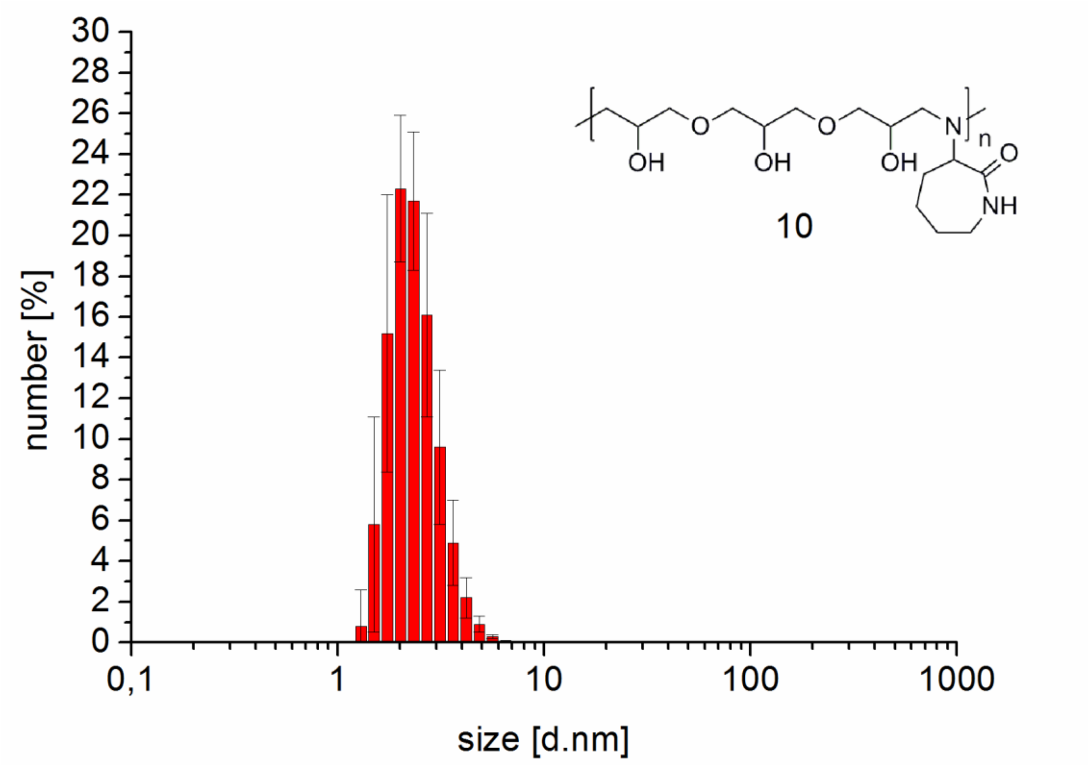

Figure S6: Mean hydrodynamic diameters of **10** in water (1.5 mg/mL) at 20 °C and standard deviation error bars.

Hysteretic effect of 10 in aqueous RAMEB-CD solution

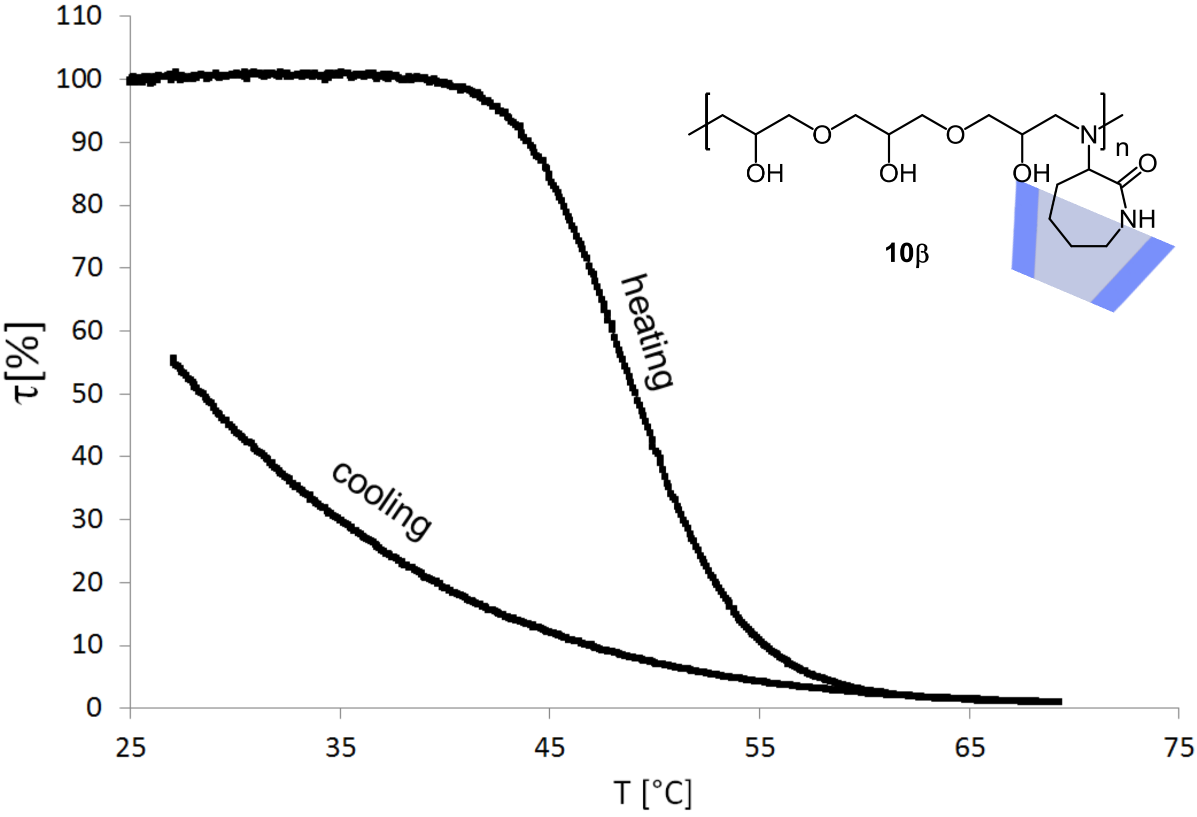

Figure S7: LCST measurements of **10** in aqueous RAMEB-CD solution with illustrated heating and cooling curve.
